# Stepwise binding of inhibitors to human cytochrome P450 17A1 and rapid kinetics of inhibition of androgen biosynthesis

**DOI:** 10.1016/j.jbc.2021.100969

**Published:** 2021-07-15

**Authors:** F. Peter Guengerich, Kevin D. McCarty, Jesse G. Chapman, Yasuhiro Tateishi

**Affiliations:** Department of Biochemistry, Vanderbilt University School of Medicine, Nashville, Tennessee, USA

**Keywords:** cytochrome P450, steroidogenesis, enzyme kinetics, enzyme inhibitor, pre–steady-state kinetics, androgen, abiraterone, ketoconazole, clotrimazole, OH, hydroxyl, P450, cytochrome P450, POR, NADPH–cytochrome P450 reductase, SVD, singular value decomposition, UPLC, ultra performance LC

## Abstract

Cytochrome P450 (P450) 17A1 catalyzes the 17α-hydroxylation of progesterone and pregnenolone as well as the subsequent lyase cleavage of both products to generate androgens. However, the selective inhibition of the lyase reactions, particularly with 17α-hydroxy pregnenolone, remains a challenge for the treatment of prostate cancer. Here, we considered the mechanisms of inhibition of drugs that have been developed to inhibit P450 17A1, including ketoconazole, seviteronel, orteronel, and abiraterone, the only approved inhibitor used for prostate cancer therapy, as well as clotrimazole, known to inhibit P450 17A1. All five compounds bound to P450 17A1 in a multistep process, as observed spectrally, over a period of 10 to 30 s. However, no lags were observed for the onset of inhibition in rapid-quench experiments with any of these five compounds. Furthermore, the addition of substrate to inhibitor–P450 17A1 complexes led to an immediate formation of product, without a lag that could be attributed to conformational changes. Although abiraterone has been previously described as showing slow-onset inhibition (*t*_1/2_ = 30 min), we observed rapid and strong inhibition. These results are in contrast to inhibitors of P450 3A4, an enzyme with a larger active site in which complete inhibition is not observed with ketoconazole and clotrimazole until the changes are completed. Overall, our results indicate that both P450 17A1 reactions—17α-hydroxylation and lyase activity—are inhibited by the initial binding of any of these inhibitors, even though subsequent conformational changes occur.

Cytochrome P450 (P450) enzymes dominate steroid metabolism (1, 2). In particular, P450 17A1 plays a central role in the conversion of the first steroid produced in the pathway from cholesterol, pregnenolone, and its 2-electron oxidation product progesterone to the 17α-hydroxy (OH) steroids needed for production of critical glucocorticoids, as well as androgens (Fig. 1). With both progesterone and pregnenolone, P450 17A1 catalyzes two NADPH-dependent and O_2_-dependent oxidations—the 17α-hydroxylation and the second, so-called “lyase” (or “desmolase”) reaction. The enzyme is important, as evidenced by >125 low-activity variants that have been identified in clinical practice (3, 4, 5). Although attenuated catalytic activity resulting in low androgen levels is one issue, inhibition is desirable under certain conditions. Huggins and Stevens (6) first demonstrated the stimulation of prostate cancer by androgens, and drugs that inhibit either the androgen receptor or androgen production are used for treatment (7).

**Figure 1:**
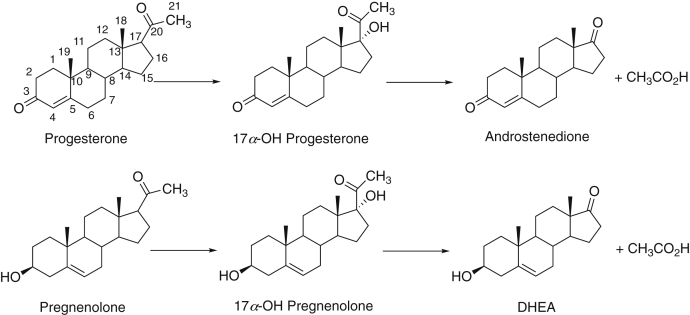
**Major reactions catalyzed by P450 17A1.** P450, cytochrome P450.

A number of drugs have been developed to inhibit P450 17A1. One of the first was ketoconazole (8, 9, 10), more generally recognized as an antifungal because of its inhibition of fungal P450 family 51 enzymes. Ketoconazole (Fig. 2), like many azoles, does inhibit other P450s. It has been used to treat prostate cancer (11), as well as fungal infections, but its inhibition of P450 3A4 (12) can give rise to serious drug–drug interactions (13, 14, 15). Other azoles, including clotrimazole (Fig. 2), have been shown to inhibit P450 17A1 (16). Some nonsteroidal azole molecules, including the naphthalene derivatives orteronel and seviteronel (Fig. 2), have been considered for their potential as drugs with P450 17A1 as a target. The pyridine-based drug abiraterone (Fig. 2), a Δ^5^-steroid, is a powerful inhibitor of P450 17A1, and used as an acetate ester prodrug (Zytiga), is the only chemical entity currently approved for use as an inhibitor of this enzyme in the treatment of prostate cancer (7).

**Figure 2:**
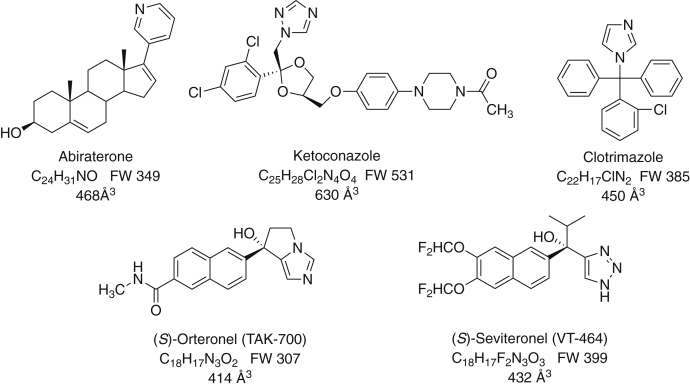
**P450 17A1 inhibitors used in this work.** The empirical formulae, formula weights, and approximate volumes of each are indicated. P450, cytochrome P450.

One of the clinical goals with P450 17A1 and androgen production in prostate cancer is the selective inhibition of the second (lyase) step relative to the first (17α-hydroxylation) in that 17α-OH steroids are needed for the synthesis of glucocorticoids (17). Abiraterone is not selective (or actually somewhat selective for 17α-hydroxylation over lyase, at least in some reports) (7). Despite some early reports (18, 19), none of the drug candidates has been very specific with regard to selectivity (7, 20, 21) (Table S1). Clinical trials for orteronel were dropped in 2016 (22) and for seviteronel in 2020 (17, 23) (although phase 2 clinical trial for orteronel in breast cancer is still listed as active (https://clinicaltrials.gov/ct2/show/NCT01990209?term=NCT01990209&draw=2&rank=1 (17))). In addition, clinical trials for another drug candidate, galeteronel (galeterone, TOK-001), were dropped in 2016 (24). The Hartmann laboratory (25) published a lead compound (an imidazole-substituted aza secosteroid) with 15-fold selectivity for lyase inhibition over 17α-hydroxylation, but to our knowledge, no further progress has been reported.

The mechanisms of these drugs have generally been considered to involve a competitive mode of inhibition, utilizing the high affinity of the heterocyclic nitrogen atoms with the P450 17A1 heme iron in the active site. This view is supported by the available X-ray crystal structures available for human P450 17A1 with bound substrates and several inhibitors (4, 20, 26). However, there is recent evidence that this may be a more complex story. The P450 17A1–orteronel X-ray structures show more than one protein conformation (20), and both NMR (27) and kinetic spectroscopic measurements (28) are consistent with the existence of multiple conformations of P450 17A1 in solution. Mixing of orteronel or seviteronel with P450 17A1 led to a series of spectral changes indicative of a multistep binding process (29). Cheong *et al.* (30) studied abiraterone inhibition of P450 17A1 and concluded that the process could be characterized as slow and tight-binding inhibition (31) (also termed slow-onset inhibition (32)), in which initial binding of an inhibitor triggers conformational changes that enhance binding and inhibition (31, 32).

In recent work with P450 3A4 and five classic and clinically important inhibitor drugs, we demonstrated a stepwise process in which the inhibitors bound to the enzyme (33), expanding on some previous kinetic studies (34). In that work, we concluded that the inhibitors did not achieve maximum inhibition until the series of steps was completed. The results may be relevant to the more general phenomenon of time-dependent inhibition commonly encountered with P450 3A4 in drug development programs (35, 36). We examined P450 17A1 reactions (Fig. 1) with ketoconazole and clotrimazole, two of the drugs used in the P450 3A4 study (33), and abiraterone, in light of the report of Cheong *et al.* (30), which indicated a *t*_1/2_ of ∼30 min for development of inhibition. We had not applied pre–steady-state kinetic assays in our previous work on inhibition of P450 17A1 by orteronel and seviteronel (29), and we have now extended the work to the lyase reaction (not only progesterone 17α-hydroxylation). Overall, the spectral and inhibition kinetics indicate multistep binding of P450 17A1 with all these inhibitors, but the results indicate that strong inhibition does not require the completion of the conformational changes.

## Results

### IC_50_ values for inhibition

**Figure 3:**
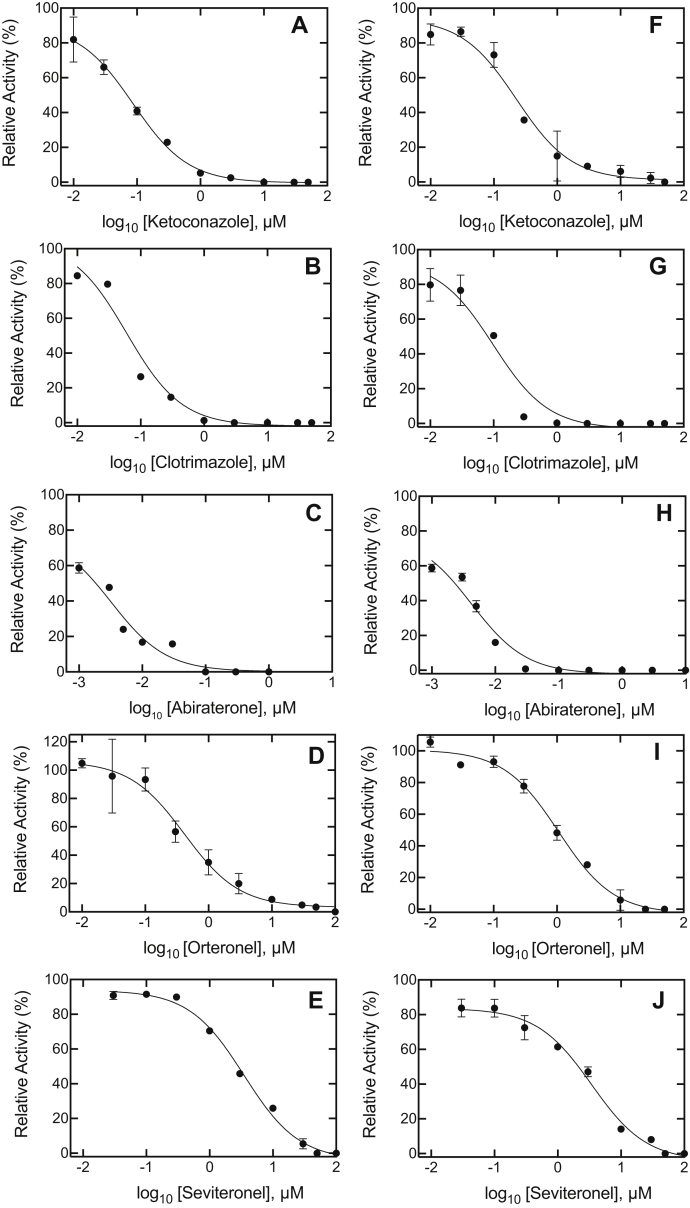
**IC**_**50**_**determinations for P450 17A1 activities.***A*–*E*, progesterone 17α-hydroxylation; *F*–*J*, 17α-OH pregnenolone lyase activity. *A* and *F*, ketoconazole; *B* and *G*, clotrimazole; *C* and *H*, abiraterone; *D* and *I*, orteronel; and *E* and *J*, seviteronel. Results are presented as means of duplicate assays. See Table 1 for values (also see Table S1 for literature comparisons). The uninhibited progesterone 17α-hydroxylation activity ranged from 4.4 to 6.0 nmol product formed min^−1^ (nmol P450)^−1^, and the 17α-OH pregnenolone lyase activity ranged from 3.1 to 5.0 nmol DHEA formed min^−1^ (nmol P450)^−1^. The *R*^2^ values ranged from 0.96 to 0.99. DHEA, dehydroepiandrosterone; P450, cytochrome P450.

Although IC_50_ values have been published for P450 17α-hydroxylation and lyase reactions (20, 21), we repeated these under our own experimental conditions (21, 37) (Fig. 3 and Table 1) before initiating more detailed kinetic studies. (Some of the studies had been done at different substrate concentrations or in cell culture.) Ketoconazole, originally developed to inhibit P450 17A1 (8, 9, 10), was a strong inhibitor of both reactions (Table 1 and Table S1). Although clotrimazole has not been used to inhibit P450 17A1 in a clinical setting to our knowledge, it has been shown to inhibit both P450 17A1 reactions (16). Abiraterone was clearly the strongest inhibitor, and even the lowest concentrations used were very inhibitory (lower concentrations would have been less than the enzyme concentration and not useful in the calculations). As pointed out in several independent studies, including our own (20, 21, 29), the selectivity of the steroid drugs in inhibiting the two reactions was not very high (Table S1).

**Table 1:** Inhibition of P450 17A1 activities: steady-state IC_50_ values

Inhibitor	IC_50_, nM (95% CI limits)^a^		Predicted *K*_i_^b^ (nM)	
	Progesterone 17α-hydroxylation	17α-OH pregnenolone lyase	Progesterone 17α-hydroxylation	17α-OH pregnenolone lyase
Abiraterone	3.2 (1.7, 6.2)	4.2 (2.6, 6.9)	1.3	3.4
Orteronel	417 (256, 680)	1060 (810, 1400)	160	870
Seviteronel	3500 (2870, 4250)	3430 (2450, 4810)	1370	2810
Ketoconazole	87 (63, 120)	227 (145, 354)	34	190
Clotrimazole	60 (37, 99)	99 (55, 176)	23	81

a From Figure 3.

b Using the relationship IC_50_ = *K*_*i*_ [1 + (S/*K*_*m*_)] for competitive inhibition, with *K*_*m*_ values from Ref. (37).

### Spectral interactions of P450 17A1 with inhibitors

**Figure 4:**
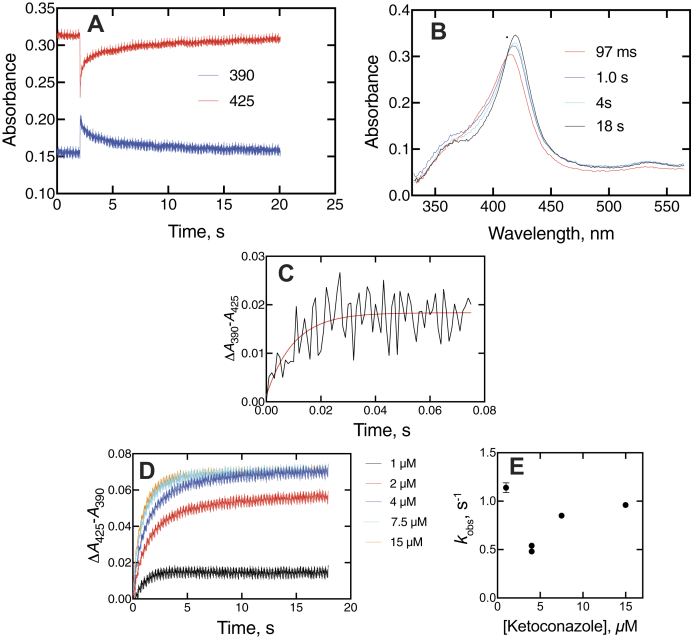
**Spectral changes observed upon mixing P450 17A1 and ketoconazole.***A*, changes in absorbance at 390 and 425 nm upon mixing 2 μM P450 17A1 and 10 μM ketoconazole (final concentrations). The instrument was used in the pretrigger mode, showing 2 s of the end of the previous reaction. *B*, spectra of complexes after mixing 2 μM P450 17A1 and 2 μM ketoconazole (final concentrations). The times after mixing are indicated. *C*, trace of early stage of changes in absorbance at 390 to 425 nm in first 80 ms after mixing. The *red line* is a fit to a first-order exponential of 100 ± 26 s^−1^. *D*, changes in absorbance at 425 to 390 nm as a function of ketoconazole concentration. *C* and *D*, the instrument was used in the same mode as in *A*, but the initial pretrigger mode data were deleted to perform fitting. *E*, plot of *k*_obs_*versus* ketoconazole (single exponential fits from *D*). P450, cytochrome P450.

**Figure 5:**
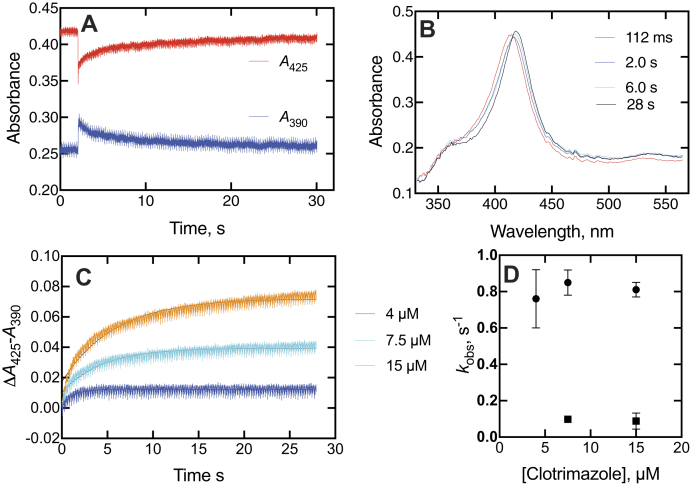
**Spectral changes observed upon mixing P450 17A1 and clotrimazole.***A*, changes in absorbance at 390 and 425 nm upon mixing 2 μM P450 17A1 and 10 μM clotrimazole (final concentrations). As in Figure 4*A*, the instrument was used in the pretrigger mode, showing 2 s of the end of the previous reaction. *B*, spectra of complexes after mixing as in *A*, with times after mixing indicated. *C*, absorbance at 425 to 390 nm traces as a function of concentration of clotrimazole (final concentrations indicated). *C*, the instrument was used in the same mode as in *A*, but the initial pretrigger mode data were deleted to preform fitting. *D*, plots of *k*_obs_ from biexponential fits (*C*) fitting to a single exponential (shown with the *lines*) were poor, and accordingly, both biexponential values were used for the analysis in *D*. P450, cytochrome P450.

Interactions between heterocyclic amines and the P450 iron atom can be useful in characterizing the affinity and kinetics. These assays were done at inhibitor concentrations higher than IC_50_ values in that higher P450 concentrations are needed for the spectroscopic studies. Both ketoconazole and clotrimazole, when mixed with P450 17A1, showed a rapid blue (hypsochromic) shift of the Soret band, followed by a slower red shift of the initial spectrum (Figs. 4 and 5) to higher wavelength in the final “type II” complex (38), as reported for P450 3A4 (33). The completion of the changes required ∼20 s in the case of ketoconazole (Fig. 4*A*). Some of the intermediate and final spectra are shown in Figure 4*B*. The rapid initial change in the spectrum upon mixing seen in Figure 4*A* for ketoconazole is expanded in Figure 4*C*, as the change in absorbance at 390 nm to absorbance at 425 nm, which occurred at a rate of ∼100 s^−1^ and peaked by 100 ms (Fig. 4*B*). Rates of the slower changes of Figure 4*A* (changes in absorbance at 425–390 nm) difference were measured at varying ketoconazole concentrations (Fig. 4*D*). The amplitude increased, up to the concentration of the enzyme, but the rates did not (Fig. 4*D*). The lack of an increase in *k*_obs_ with the ligand concentration is further evident for the domination of a conformational selection mechanism for the slow binding changes, a conclusion reached earlier with substrates (28) and the inhibitors abiraterone (28) and orteronel (29).

Clotrimazole yielded similar results as ketoconazole (Fig. 5). The biphasic changes in the spectra were also seen, requiring nearly 30 s for completion (Fig. 5*A*). Similar intermediate spectra were observed (Fig. 5*B*). Although clotrimazole was a slightly better inhibitor than ketoconazole, as judged by the IC_50_ results (Fig. 3, *A*, *B*, *F*, and *G* and Table 1), the spectral changes were not as pronounced as with ketoconazole, and higher concentrations were required (Fig. 5*C*). These traces did not fit well to single exponential plots, but plots of the individual biexponential *k*_obs_ values *versus* clotrimazole concentration did not lead to an increase in *k*_obs_ (Fig. 5*D*), and the conclusion is also that the process is also dominated by conformational selection (28, 39, 40).

Three other P450 3A4 inhibitors that we studied previously (33)—itraconazole, ritonavir, and indinavir—did not show strong enough spectral interaction with P450 17A1 to pursue these studies. (These were not tested for inhibition of enzyme activity.)

**Figure 6:**
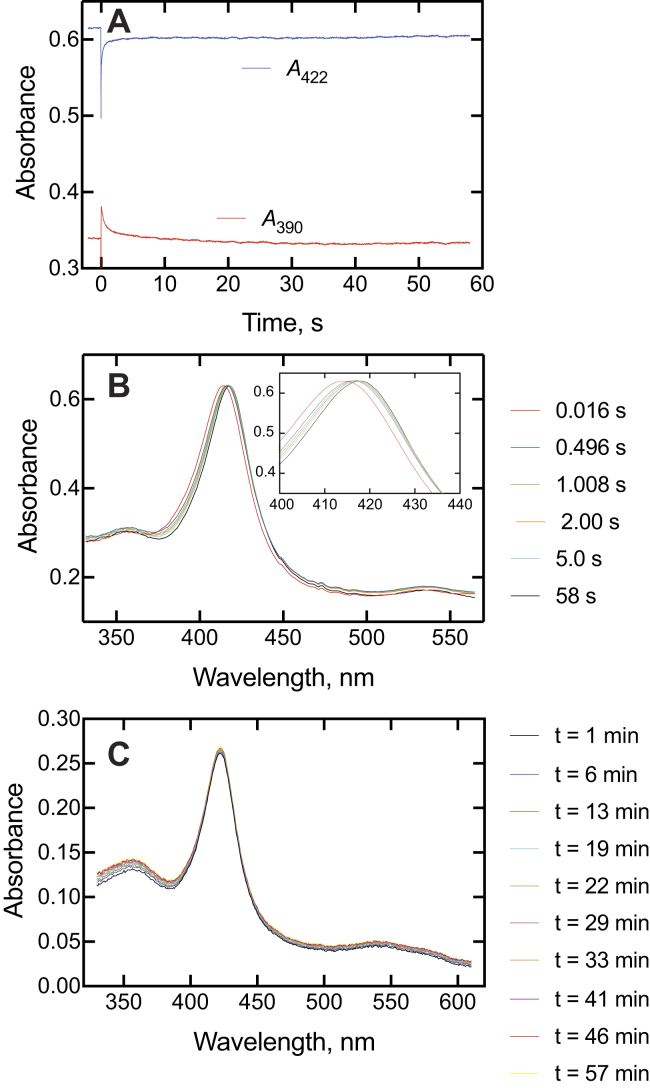
**Spectral changes observed upon mixing P450 17A1 and abiraterone.** P450 17A1 (2 μM) and abiraterone (2 μM) were mixed. *A*, changes in absorbance at 390 and 422 nm over 60 s. As in Figures 4*A* and 5*A*, the instrument was used in the pretrigger mode, showing 2 s of the end of the previous reaction. In this case, the zero time point is corrected. *B*, intermediate spectra collected 16 ms to 56 s after mixing. An expansion of the Soret peak is shown in the inset, with the early (16 ms) spectrum, followed by the isosbestic change from the 496 ms spectrum to the final complex (58 s). *C*, spectra collected from 1 to 57 min after mixing. P450, cytochrome P450.

Some spectral binding studies with P450 17A1 and abiraterone had been presented previously (28) and interpreted in the context of a conformational selection model (as opposed to induced fit). More studies (Fig. 6*A*) showed that the spectral changes were similar to what had been seen with ketoconazole and clotrimazole, with intermediate spectra observed over a period of ∼5 s and the final complex at 58 s (Fig. 6*B*). Although abiraterone has been described as a slow and tight-binding inhibitor with a *t*_1/2_ of ∼30 min for conversion to an inhibitory complex (30), no further spectral changes were observed over a period of 1 h (Fig. 6*C*).

**Figure 7:**
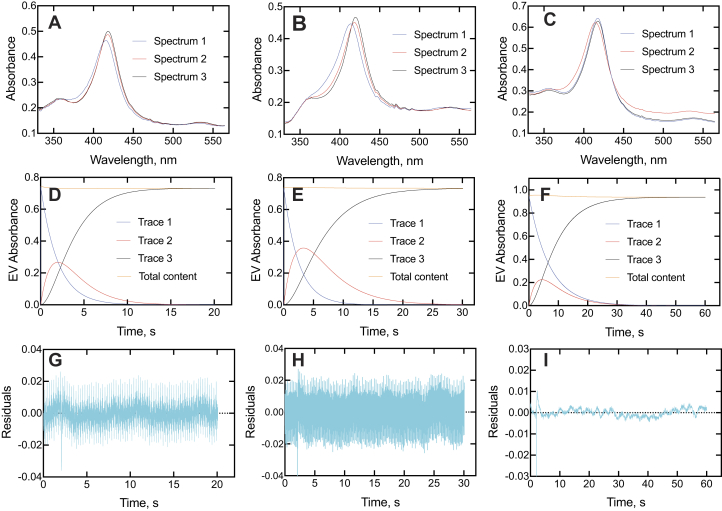
**SVD analyses of binding of ketoconazole, clotrimazole, and abiraterone to P450 17A1.***A*–*C*, SVD spectra of P450 17A1 complexes following an initial spectrum (spectrum 1) for ketoconazole, clotrimazole, and abiraterone, respectively. *D*–*F*, time course of changes in SVD spectra (*A*–*C*) for ketoconazole, clotrimazole, and abiraterone, respectively. The *blue lines* (trace 1) show the loss of the initial spectrum 1, *red lines* (trace 2) show the course of the appearance and disappearance of spectrum 2, and *black lines* (trace 3) show the appearance of the final complex (spectrum 3). The nearly *horizontal red lines* at the tops of *D*–*F* indicate the total content of spectral species mathematically accounted for during the time courses. *G*–*I*, residual analysis for *G*, *H*, and *I* for ketoconazole, clotrimazole, and abiraterone, respectively. P450, cytochrome P450; SVD, singular value decomposition.

Singular value decomposition (SVD) spectra are useful in that they are based on all spectral data points and not biased by the selection of individual wavelengths. These were consistent with the presence of at least three distinct spectral complexes (Fig. 7, *A*–*C*), in general agreement with the trends of the actual spectra (Figs. 4*B*, 5*B*, and 6*B*). It should be pointed out that the SVD procedure is designed to detect a minimum of changes that occur, though, and the actual spectra are indicative of a more complex reaction (Figs. 4*B*, 5*B*, and 6*B*). With all three inhibitors, a transient SVD peak was maximal at ∼ 2 s (Fig. 7, *D*–*F*). The abiraterone spectra are somewhat different from those observed with ketoconazole (Fig. 5*B*), clotrimazole (Fig. 6*B*), seviteronel, and orteronel (29) in that the second complex is the one with the largest blue shift (spectrum 2 in Fig. 7*D*). Overall, all the SVD spectra indicate that the slow formation of the spectral complexes is multiphasic, regardless of how many steps are actually discriminated. There were attempts to use only two-state SVD to describe the data were unsuccessful as judged by the poor fits of the residuals, which were well clustered along the *x*-axis in the SVD fits shown (Fig. 7, *G*–*I*).

### Rescue of catalytic activity from inhibitors

In these experiments, a 1:1 M complex (EI) of P450 17A1 (E) and inhibitor (I) was mixed with NADPH plus an excess of substrate (S) to initiate the reaction to form the product P. The concept is that first-order release of free E is needed to allow binding of S, that is,

(∗EI⇄)EI⇄I+E

E+S→ES→EP→E+P

where ∗EI, if present, is a conformationally distinct EI complex. All assays were done at 23 °C (instead of 37 °C) to minimize any enzyme denaturation during the incubation period. The method can provide evidence for the slow conversion of ∗EI to EI, if this is slow (32).

**Figure 8:**
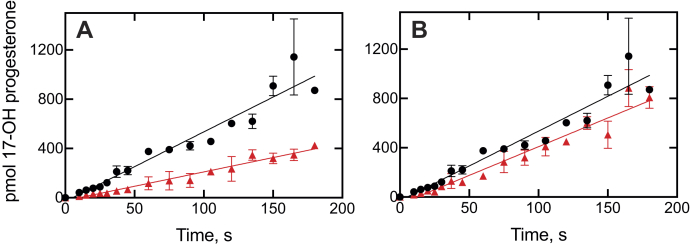
**Kinetics of recovery of 17α-hydroxylation activity from P450 17A1–inhibitor complexes upon addition of progesterone.***A*, ketoconazole; *B*, clotrimazole. The incubations contained equal concentrations (250 nM) of P450 17A1 and the inhibitor, and the reactions were initiated by the addition of the NADPH-generating system supplemented with progesterone (20 μM). The data were fit to linear equations for the uninhibited (●, *black lines*) and inhibited (▲, *red lines*) reactions. The uninhibited rate of progesterone 17α-hydroxylation was 0.045 s^−1^ (*i.e.*, 2.7 pmol product formed min^−1^ [pmol P450 17A1]^−1^). *R*^2^ values ranged from 0.91 to 0.93. P450, cytochrome P450.

**Figure 9:**
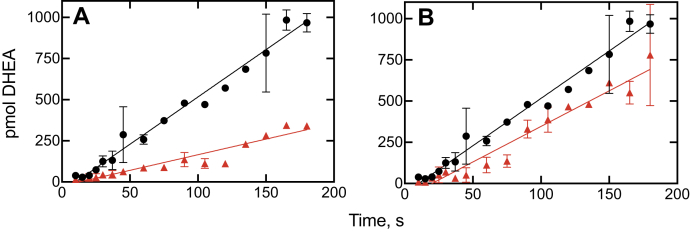
**Kinetics of recovery of lyase activity from P450 17A1–inhibitor complexes upon addition of 17α-OH pregnenolone.***A*, ketoconazole; *B*, clotrimazole. The incubations contained equal concentrations (250 nM) of P450 17A1 and the inhibitor, and the reactions were initiated by the addition of the NADPH-generating system supplemented with 17α-OH pregnenolone (20 μM). The data were fit to linear equations for the uninhibited (●, *black lines*) and inhibited (▲, *red lines*) reactions. The uninhibited rate of DHEA formation was 0.046 s^−1^ (*i.e.*, 2.8 pmol product formed min^−1^ [pmol P450 17A1]^−1^). *R*^2^ values ranged from 0.89 to 0.95. DHEA, dehydroepiandrosterone; P450, cytochrome P450.

Experiments were done with the inhibitors ketoconazole and clotrimazole, using a short time scale for the kinetic analysis (Figs. 8 and 9). The results showed that the inhibition plots could be fit to linear plots for both P450 17A1-catalyzed progesterone 17α-hydroxylation (Fig. 8, *A* and *B*) and 17α-OH pregnenolone lyase (Fig. 9, *A* and *B*) reactions. Any suggestion of lags is no greater than in the uninhibited reactions.

A lag phase in the aforementioned rescue experiments would be consistent with the need for a conformational change associated with enzyme release and binding but would not necessarily prove its existence, as has been pointed out earlier (33). A very tightly bound inhibitor can also show such lag phases in simple models, for example, Figures 2 and 3 of Ref. (41).

### Rates of onset of inhibition

**Figure 10:**
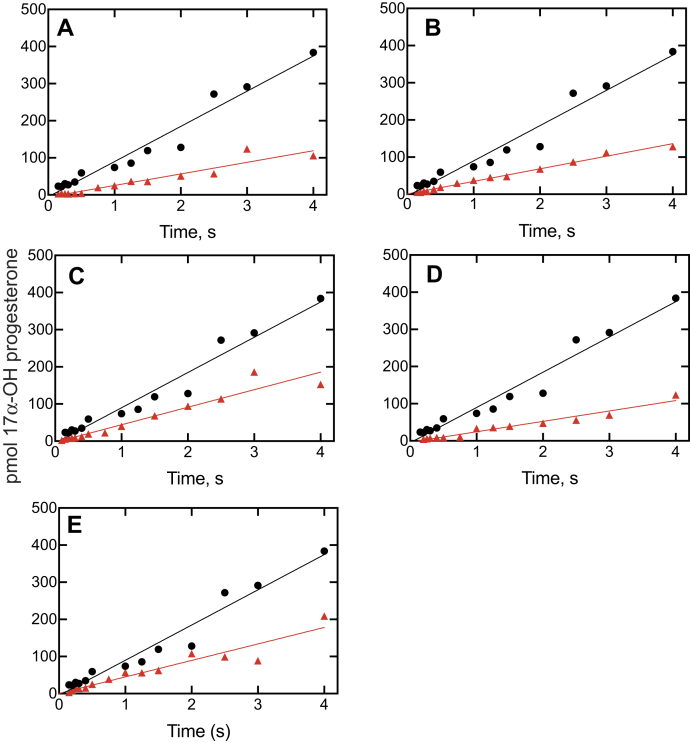
**Kinetics of inhibition of P450 17A1-catalyzed progesterone 17α-hydroxylation.** Plots of formation of 17α-OH progesterone are shown for no inhibitor (●, *black lines*) and for: *A*, ketoconazole (1 μM); *B*, clotrimazole (1.5 μM); *C*, seviteronel (3 μM); *D*, orteronel (1.5 μM); and *E*, abiraterone (1 μM). The P450 concentration was 2 μM, and the (final) inhibitor concentrations are indicated. The data were fit to linear equations for the uninhibited (●, *black lines*) and inhibited (▲, *red lines*) reactions. The uninhibited rate of progesterone 17α-hydroxylation was 0.12 s^−1^ (*i.e.*, 7.2 pmol product formed min^−1^ [pmol P450 17A1]^−1^). *R*^2^ values ranged from 0.91 to 0.99. P450, cytochrome P450.

**Figure 11:**
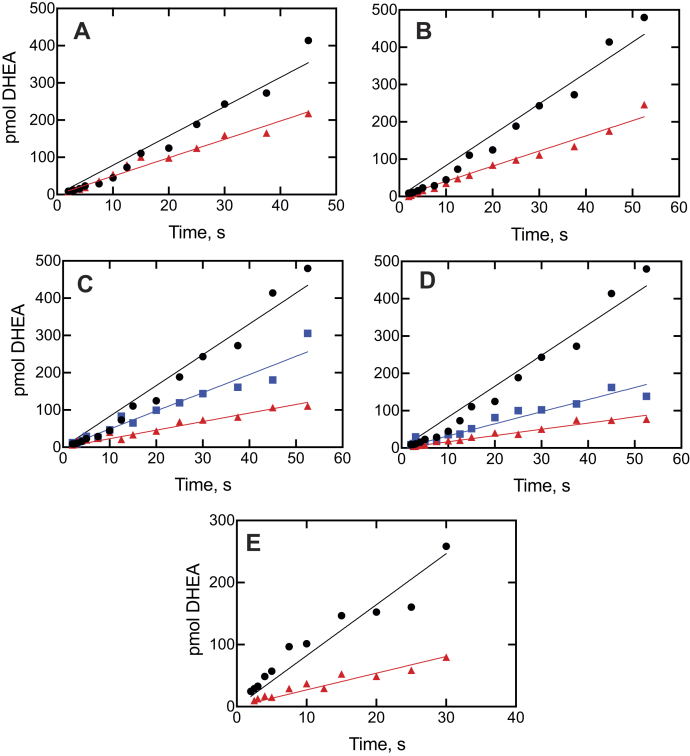
**Kinetics of inhibition of P450 17A1-catalyzed 17α-OH pregnenolone lyase activity.** Plots of conversion of 17α-pregnenolone to DHEA are shown for no inhibitor (●, *black lines*) and for: *A*, ketoconazole (1 μM, ▲, *red line*); *B*, clotrimazole (1.5 μM, ▲, *red line*); *C*, seviteronel (1.5 μM, ▲, *blue line* and 15 μM, ▲, *red line*); *D*, orteronel (1.5 μM, ▲, *blue line* and 5 μM, ▲, *red line*); and *E*, abiraterone (1 μM, ▲, *red line*). The P450 concentration was 2 μM, and the (final) inhibitor concentrations are indicated. The data were fit to linear equations. The uninhibited rate of DHEA formation was 0.0092 s^−1^ (*i.e.*, 0.55 pmol product formed min^−1^ [pmol P450 17A1]^−1^). *R*^2^ values ranged from 0.935 to 0.98. DHEA, dehydroepiandrosterone; P450, cytochrome P450.

This experimental design differs from the previous section in that an ES complex is mixed with I and the time course of product is measured, that is, ES ⇄ S + E ⇄ EI ⇄ E∗I, providing a more rigorous analysis of slow onset inhibition. In the control experiment, ES is not inhibited (no I present). If a conformational change is required after binding I to E to achieve full inhibition, then there should be an exponential phase preceding the (final) inhibited steady state (32, 33). Both progesterone 17α-hydroxylation (Fig. 10) and 17α-OH pregnenolone lyase activity (Fig. 11) were measured without and with all five inhibitors.

A pre–steady-state rapid chemical quench time frame was used (33), in order to evaluate subsecond inhibition (Figs. 10 and 11). All the inhibition plots fit well to both linear plots, that is, *R*^2^ values of 0.91 to 0.99. Fits to log-linear biphasic kinetics were no better than linear, implying that the inhibition was occurring immediately after mixing and not requiring protein changes.

### Abiraterone inhibition kinetics

With the drug abiraterone, an earlier report calculated *k*_off_ to be 0.0093 min^−1^ (*t*_1/2_ = 5 h) (42) and another reported the *t*_1/2_ for the onset of inhibition to be ∼30 min (30). Our initial experiments indicated that inhibition was seen rapidly (Figs. 3, *C* and *H*, 10*E*, and 11*E*). In order to preclude any issues (*e.g.*, with P450 17A1 stability during the preincubation period) and to provide direct comparisons with the previous work (30), we utilized the same commercially available reagent, that is, *Escherichia coli* membranes in which P450 17A1 and NADPH–cytochrome P450 reductase (POR) had been coexpressed (Cypex Bactosomes). A linear dependence of product formation on P450 concentration was established (Fig. S7), and the (exogenous) *b*_5_ concentration was optimized (Fig. S8). Although the P450 and POR are expressed in the membranes, most of each of the two proteins is apparently exposed, and the same strong dependence of lyase activity on exogenous *b*_5_ was seen as in the case of the reconstituted enzyme system (21, 37). As observed before in the reconstituted systems (21, 37), *b*_5_ only stimulated progesterone 17α-hydroxylation twofold and/or lesser.

**Figure 12:**
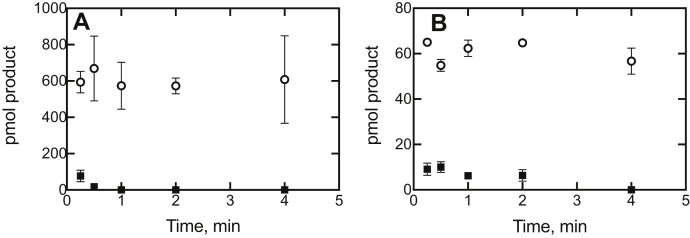
**Kinetics of inhibition of P450 17A1-catalyzed activity as a function of preincubation time with abiraterone.***A*, progesterone 17α-hydroxylation; *B*, 17α-OH pregnenolone lyase activity (to form DHEA). These experiments utilized bacterial membranes (CYP17A1R Bactosomes) as the source of P450 and POR. Experiments were done with 10 nM P450 17A1 in reaction volumes of 0.5 ml, with 100 nM *b*_5_ added. Abiraterone was added to 50 nM, and then, the reactions were initiated by the addition of an NADPH-generating system supplemented with either 20 μM progesterone (*A*) or 17α-OH pregnenolone (*B*) at the indicated times and proceeded for 5 min (at 37 and 23 °C, respectively). Reactions were done in duplicate, and the results are shown as means ± SD (range): no inhibitor (◯); plus 50 nM abiraterone (■). The uninhibited rates of (*A*) 17α-OH progesterone and (*B*) DHEA production were 24 and 2.4 pmol formed min^−1^ (pmol P450 17A1)^−1^, respectively. DHEA, dehydroepiandrosterone; P450, cytochrome P450; POR, NADPH–cytochrome P450 reductase.

The results of an experiment in which the preincubation time with abiraterone was varied showed that progesterone 17α-hydroxylation (Fig. 12*A*) and 17α-OH pregnenolone lyase (Fig. 12*B*) activities were almost completely inhibited after 15 s, which is consistent with the rapid onset of inhibition seen in Figures 10*E* and 11*E*.

**Figure 13:**
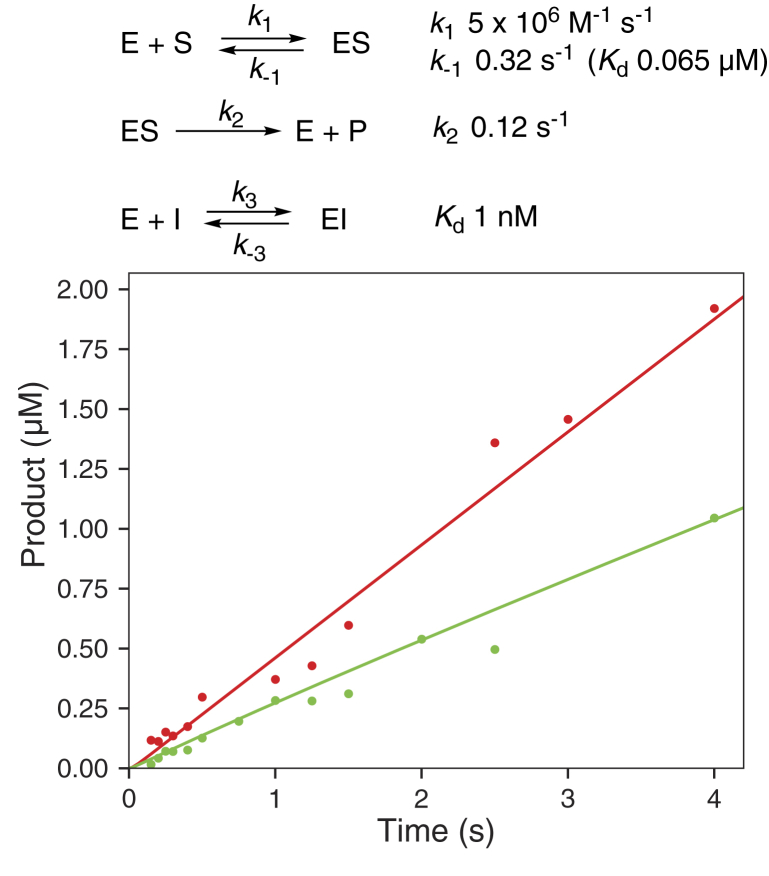
**Modeling of kinetics of inhibition of P450 17A1-catalyzed progesterone 17α-hydroxylation activity by abiraterone.** The data points are from Figure 10*E*. The lines were fit to the model shown, with the indicated rate and dissociation constants. The enzyme concentration (E) was 2 μM, substrate concentration (S) was 5 μM, and inhibitor concentration (I, abiraterone) was 1 μM. Fitting was done with KinTek Explorer. P450, cytochrome P450.

The results of the progesterone 17α-hydroxylation abiraterone inhibition experiment in Figure 10*E* were analyzed further using KinTek Explorer software (KinTek; Fig. 13). A simple mechanism with a progesterone *K*_*d*_ of 0.065 μM we reported in a recent study (37) and a *K*_*d*_ of 1 nM for abiraterone, as a competitive inhibitor, sufficed to provide adequate fits, without any obvious need to use a more complex model. It should be noted that inhibition was noted already in the earliest time points (≤100 ms).

## Discussion

The main features of the spectral changes observed upon binding of ketoconazole, clotrimazole, and abiraterone to P450 17A1 were similar to those we previously reported with orteronel and seviteronel in a study involving only progesterone 17α-hydroxylation (29). The studies with seviteronel and orteronel (29) were extended to P450 17A1 17α-OH pregnenolone lyase reactions and the use of pre–steady-state kinetics for both the P450 17A1 reactions. However, we reached the same conclusion as in the preliminary work with orteronel and seviteronel (29), that is, that inhibition of (both) P450 17A1 reactions did not require completion of all the P450 17A1 changes that were observed spectrally. This was also the case with ketoconazole and clotrimazole. Although abiraterone has been reported to be a slow and tight-binding (or “slow onset”) inhibitor of P450 17A1 (30), it also fits into this category with the other inhibitors in terms of not requiring time to develop.

Our IC_50_ values can be compared with others for human P450 17A1 reactions in the literature (Table S1). The values show considerable interstudy variation. Some of this variation is due to the fact that IC_50_ values are dependent upon experimental conditions, particularly substrate concentration and ratios of the three individual enzymes (P450 17A1, POR, and *b*_5_), and are not as informative as *K*_*i*_ values (which are also estimated in Table 1). With a few exceptions, our IC_50_ values are as low or lower. The main interest is the selectivity for the lyase reaction (Fig. 1), reflected in the ratio of IC_50_ values for progesterone 17α-hydroxylation:17α-OH pregnenolone lyase activities. Although some reports of high selectivity have appeared, we did not obtain any values greater than unity in the current study (Table 1 and Table S1), and only a few high values have been reported by developers of particular drug candidates (Table S1). The only P450 17A1 inhibitor drug currently on the market, abiraterone, does not have much selectivity for the lyase reaction, as reported by others (7, 20, 25).

The spectral changes observed for binding of the inhibitors were similar (Figure 4, Figure 5, Figure 6, Figure 7). The development of type II binding spectrum was much too slow to be a diffusion-limited process, as seen in the case of P450 3A4 (33, 34), and we investigated aspects of a multistep process, as already reported for orteronel and seviteronel (29). In every case, there was rapid binding and a blue (hypsochromic) shift to lower wavelength, followed by what appear to be two changes leading to the final complex (Figs. 4*B*, 5*B*, and 6*B*), with the conclusion supported by SVD analysis of the accumulated spectra (Fig. 7). Conformational selection dominates in the binding of steroids to P450 17A1 (28), indicating multiple conformations of P450 17A1 in the absence of ligands. On the basis of these results, the structural work (20), and the other evidence accumulated here with drugs (Figs. 4*E* and 5*D*), we conclude that the equilibria for P450 17A1 are at least as complex as shown:

E+S⇆ES

$${\text{E}}^{‡}⇆\text{E}+\text{I}⇆\text{EI}⇆{\text{E}}^{\prime }\text{I}⇆\text{E}*\text{I}$$

where E, E^‡^, E′, and E∗ are conformationally distinct forms of E (I is an inhibitor). Only free E can bind the substrate S, and this competition is the basis for the inhibition (28). This is consistent with the X-ray crystal structures of P450 17A1 with ligands, which generally appear not to allow space for simultaneous occupancy by a substrate and inhibitor (4, 20, 26). Binding of a second inhibitor at a peripheral site has been observed for (*S*)-orteronel, between the F/G helices and the N terminus (20) (but not for (*R*)-orteronel, which is also inhibitory (20, 21)). At this time, we cannot totally dismiss the possibility of both a substrate and an inhibitor being bound at the same time, but our evidence suggests that this is not occurring. Even if it does happen, it does not prevent rapid inhibition.

The kinetics of interaction of substrates and inhibitors with P450 17A1 can be compared, based on previous studies (21, 28, 29) and this work (Figure 4, Figure 5, Figure 6, Figure 7, Figure 8, Figure 9, Figure 10, Figure 11, Figure 12, Figure 13). The initial binding of both substrates and inhibitors to P450 17A1 is rapid, that is, on the order of 10^6^ M^−1^ s^−1^ (21, 28, 29). The first step in binding ketoconazole was also rapid (Fig. 4*C*). In the case of substrate binding (28), the initial binding was followed by spectral changes with first-order rates of 5 to 10 s^−1^ and 0.8 to 1.0 s^−1^, arriving at a predominantly high-spin Soret peak (λ_max_ = 390 nm). With the inhibitors, initial binding yielded a Soret peak indicative of partial high-spin character (Figs. 4*B*, 5*B*, and 6*B*) (29). This shifted to a second intermediate at a rate of 1 to 3 s^−1^ (28, 29) (Figs. 4, *D* and *E* and 5, *C* and *D*) and then to the final low-spin (type II) complex at a rate of ∼0.1 s^−1^ (28) (Fig. 5*D*). For comparison, steady-state rates of progesterone 17α-hydroxylation and 17α-OH pregnenolone lyase activity were 0.05 to 0.1 s^−1^ (Figure 8, Figure 9, Figure 10, Figure 11 and 13). These are only about as fast as the final steps of the oxidation reactions and may raise questions about the relevance of the inhibitor studies. However, rapid chemical-quench pre–steady-state kinetic assays (single turnover) showed that the actual rate constants for substrate oxidation reactions were faster, that is, 0.08 to 0.47 s^−1^ (21). The modeling in Figure 13 used a rate constant (*k*_2_) of 0.12 s^−1^ (*i.e.*, 7.2 min^−1^ for product formation [at 23 °C]). The most relevant results in this regard are probably those in Figures 10 and 11, in which inhibition of product formation is observed already in less than 1 s, within the time frame of a single enzyme cycle. A simple enzyme kinetic model (Fig. 13) supports our view that this basic scheme can account for the observed results.

Abiraterone has been reported to inhibit P450 17A1 with “mixed” inhibition-type steady-state kinetics (30, 42). In principle, mixed inhibition involves the formation of a complex of both substrate and inhibitor with an enzyme (43), which seems unlikely given what is known about the structures of the P450 17A1 substrate (26) and abiraterone (4) (and an analog) (44) complexes (with the case of (*S*)-orteronel mentioned earlier (20)). However, this term (mixed inhibition) is widely invoked in the absence of mechanistic information and is often based on limited kinetic data points (30, 42), in the absence of other physical data.

What is not clear about some of the previous work with abiraterone is why some studies reported time-dependent inhibition (30, 45), which was clearly not seen in our own work (Figure 10, Figure 11, Figure 12, Figure 13) or that of others (4, 17, 20, 42). The inhibition occurred (with the bacterial membrane preparations) within less than 1 s (Figs. 10, 11, and 13).

The results can be contrasted with our work on P450 3A4 (33), where full inhibition was not realized until the course of spectral changes was completed. With P450 17A1, inhibition began immediately (Figure 8, Figure 9, Figure 10, Figure 11, Figure 12, Figure 13). We propose that P450 17A1 moves through multiple conformations observed with the spectral changes (Figure 4, Figure 5, Figure 6, Figure 7), or at least the inhibitor is moving within the active site to cause the changes. Why is the nature of inhibition different from P450 3A4? Perhaps, the most likely reason is simply the size of the enzyme active site. The available structural information for human P450 17A1 is that only one steroid molecule or inhibitor can be accommodated (4, 20, 26), with the possible exception of the peripheral (*S*)-orteronel binding mentioned earlier (20). However, the active site of P450 3A4 is much larger (46) and can bind two molecules of ketoconazole (47) or a ritonavir analog (48), and a binding site removed from the canonical active site has been reported at least twice (49, 50). It would seem very reasonable to expect complexes of P450 3A4 to contain molecules of both substrate and inhibitor, although none have been reported to our knowledge. The size of the canonical active site (∼1400 Å^3^) (46) also allows for more tumbling of ligands than P450 17A1 (Fig. 2), which is a much more selective enzyme.

In summary, we are left with an evolving picture of P450s that undergo conformational changes, both with and without ligand bound. Some of these changes are related to enhance binding of substrates and inhibitors, but what occurs with one P450 may or may not apply to others.

## Experimental procedures

### Chemicals

Progesterone, 17α-OH pregnenolone, ketoconazole, clotrimazole, dansyl hydrazine, and 1,2-α-dilauroyl-*sn*-glycero-3-phosphocholine were purchased from Sigma–Aldrich. (*S*)-Seviteronel was purchased from Advanced ChemBlocks, and its purity was characterized previously (29). Purified (*S*)-orteronel was purchased from AOBIOUS. Abiraterone was obtained from Selleckchem, and its purity was previously determined (28).

### Enzymes

Slightly modified versions of human P450 17A1 (21, 51), human *b*_5_ (52), and rat POR (53) were expressed in *E. coli* and purified to near electrophoretic homogeneity using the cited procedures. Some of the experiments with abiraterone were done with commercial CYP17A1R Bactosomes (high reductase), which *E. coli* membranes containing expressed human P450 17A1 and POR (Cypex/Sekisui XenoTech), in order to replicate conditions used in a published study (30) (purified recombinant human *b*_5_ was added to these for the lyase reactions, Fig. S8).

### Steroid derivatization

The procedure is based on a method involving formation of dansyl hydrazones (Fig. S1) (54, 55).

In the case of catalytic assays, the products of each reaction were redissolved in a solution of CF_3_CO_2_H (7 mM, prepared as a 0.1% v/v solution in C_2_H_5_OH) and dansyl hydrazine (10 mM, prepared in CH_3_OH) in amber glass vials (sealed and capped) and were allowed to react overnight (16 h) in the dark at room temperature (23 °C). The reaction was quenched with acetone (50 mM) followed by incubation at room temperature for 30 min. NaOH was added to 100 mM, and the products of the reactions were extracted into CH_2_Cl_2_ (1.0 ml), of which 0.8 ml of the organic (lower) layers was transferred to fresh amber vials and dried under a stream of nitrogen. The residues were redissolved in a mixture of a 0.1% HCO_2_H solution (aqueous) and CH_3_CN (0.1 ml; 1:1 dilution, v/v) and transferred to ultra performance LC (UPLC) vials for analysis. Although LC fluorescence could be used for analysis (Fig. S2), we found that LC–MS using positive-ion electrospray (even with a single quadrupole instrument) was much more sensitive (Fig. S3). Samples (held at 25 °C) were injected (10 μl) on a Waters Acquity UPLC (40 °C) using a 2.1 × 50 mm Acquity BEH octadecylsilane (C_18_) column (1.7 μm) and were separated at a flow rate of 0.2 ml min^−1^ using a gradient of solutions of (A) 0.1% aqueous HCO_2_H and (B) CH_3_CN as follows (all v/v): 0 min, 60% A; 0.5 min, 60% A; 8 min, 0% A; 8.5 min, 5% A; 9 min, 0% A; 9.1 min, 60% A; and 10 min, 60% A. Samples were detected with an online mass spectrometer (Waters QDa Detector, positive-ion mode) using a cone voltage of 15 V, a sampling frequency of 10.0 Hz, and scanning from *m/z* 150 to 800. Data were processed using MassLynx software (Waters) (Fig. S5). The standard curves were linear over a range of 0.01 to 10 pmol dehydroepiandrosterone (Fig. S6).

Hydrazones exist as *E* and *Z* isomers (Fig. S1). Analysis of the products by UPLC showed multiple peaks (Fig. S2), but all these had the expected MH^+^ peaks, indicating that they were the expected isomers. Furthermore, the (di) dansyl hydrazone derivative of 17α-OH progesterone was isolated, and its ^1^H NMR spectrum showed the characteristic two peaks expected for the H-4 proton of the steroid A ring (δ 5.73, 6.07), in a 2:1 ratio (Fig. S4). Other NMR peaks were consistent with the structure. Accordingly, we summed the integrals of all isomeric product peaks (Fig. S5) in constructing the standard curves (Fig. S6) and in the analysis of the products (Fig. S7).

### IC_50_ determinations

#### 17α-OH progesterone formation

A 0.5 ml reaction was prepared in most cases by the reconstitution of P450 17A1 (0.02 μM), POR (0.5 μM), and *b*_5_ (0.5 μM) with freshly sonicated L-α-dilauroyl-*sn*-glycero-3-phosphocholine (30 μM). The mixture incubated on ice for 10 min, followed by the addition of potassium phosphate buffer (to a final concentration of 50 mM), substrate (5 μM progesterone or 1.5 μM 17α-OH pregnenolone (37)), and water. Stock progesterone was prepared in CH_3_OH and was diluted to a final concentration of 5 μM (0.5% CH_3_OH, v/v). The inhibitors ketoconazole, clotrimazole, abiraterone, (*S*)-orteronel, and (*S*)-seviteronel were prepared in CH_3_OH and added to incubations at 0.5%, v/v (0–100 μM), bringing the final CH_3_OH composition to 1% (v/v) in the reaction. Reactions (in duplicate) were preincubated for 5 min at 37 °C with shaking before being initiated with an NADPH-generating system composed of 0.5 mM NADP^+^, two units of yeast glucose 6-phosphate dehydrogenase ml^−1^, and 10 mM glucose 6-phosphate (56). The inhibitor abiraterone was added immediately prior to the 5-min preincubation stage because of the rapid onset of inhibition. Reactions (5 min) were quenched by immersion in an ice bath, with the addition of CH_2_Cl_2_ (2.0 ml), and were centrifuged (10^3^*g*, 10 min) to separate layers. The organic (bottom) phases were removed (1.6 ml) and transferred into clean vials, and the solvent was removed under a stream of nitrogen. Reactions were resuspended in a CH_3_CN/sodium acetate mixture (25 mM, pH 3.7) (1:1, v/v) and were transferred to vials for UPLC analysis. Samples (held at 4 °C) were injected (15 μl) on a Waters Acquity UPLC (25 °C) using a 2.1 × 100 mm Acquity BEH octadecylsilane (C_18_) column (1.7 μm) and were separated using an isocratic mobile phase of 25 mM acetate buffer (pH 3.7) and CH_3_CN (4:6, v/v) at a flow rate of 0.2 ml min^−1^ (57). Substrate and product were detected using a Waters Acuity photodiode array system at 240 nm. Data were processed using the MassLynx software, and percent conversion of substrate to product was calculated. The data were normalized to the uninhibited reaction, which was set at 100% activity.

#### Dehydroepiandrosterone

Experimental conditions for the lyase reaction were identical to the hydroxylation reaction with the following exceptions: 17α-OH pregnenolone (1.5 μM) was used as the substrate, and following extraction, the product of the reaction was derivatized with dansyl hydrazine as described previously.

### Steady-state kinetic inhibition assays

Steady-state kinetic inhibition assays were performed using the same basic reconstituted system as described for the IC_50_ determinations but with the concentration of P450 17A1 increased to 250 nM. The reconstitution was then preincubated with an equimolar (250 nM) amount of inhibitor (ketoconazole, clotrimazole, (*S*)-seviteronel, or (*S*)-orteronel) at room temperature (23 °C) before initiation with a NADPH-generating system (prepared as previously described) that was supplemented with either 17α-OH pregnenolone or progesterone (20 μM). Reactions (10–180 s) were quenched with CH_2_Cl_2_ (2.0 ml) and chilled on ice. The products of both reactions then followed the steroid derivatization procedure where they were centrifuged, extracted, and derivatized with dansyl hydrazine for LC–MS detection.

Steady-state kinetic inhibition assays performed with Cypex CYP17A1R Bactosomes followed largely the same procedure with the following exception: the enzymatic system was prepared by preincubating (23 °C) P450 17A1 (CYP17A1R Bactosomes; 10 nM P450), *b*_5_ (100 nM), and potassium phosphate buffer (50 mM, pH 7.4) with abiraterone (50 nM) for varying lengths of time (0.25–30 min). Reactions (5 min) were then initiated with the NADPH-generating system described previously and subjected to the same procedure.

### Pre–steady-state kinetic assays (activity)

The same basic enzyme reconstitution was used for the kinetic inhibition assays as previously described for the IC_50_ determinations but with the concentrations of P450 17A1, *b*_5_, and POR increased several fold (4, 4, and 8 μM, respectively). Reactions were performed using a KinTek RQF-3 rapid quench apparatus (KinTek) with the reaction loop set at position 7 and the temperature at 37 °C. The RQF-3 is a rapid mixing device that initiates a reaction by forcing equal volumes of two mixing syringes into a reaction loop. After pausing for the indicated incubation time, the reaction is then quenched and expelled from the apparatus. The reaction mixture (containing enzyme and substrate [in CH_3_OH, 1% (v/v)]) was initiated with an equal volume (19 μl) of NADPH solution (2 mM), effectively halving the initial concentration of all reaction components. When appropriate, inhibitor (in CH_3_OH) was added to the NADPH solution (in CH_3_OH), taking care to keep the total CH_3_OH composition of the final reaction to 1% (v/v). The substrates progesterone (5 μM) and 17α-OH pregnenolone (1.5 μM) were allowed to react for different lengths of time (0.1–5 and 2–60 s, respectively) prior to quenching with 160 μl of 1 M HCl. Five replicates of each time point were collected into vials to increase the detection sensitivity of the respective product at the shorter time points. The products of both reactions then followed the steroid derivatization procedure where they were centrifuged, extracted, and derivatized with dansyl hydrazine for LC–MS detection.

### Spectroscopy

Measurements of P450, *b*_5_, and POR were made with an OLIS-Aminco DW2 spectrophotometer (On-Line Instrument Systems). Pre–steady-state kinetic assays of binding of inhibitors were made using an OLIS RSM 1000 stopped-flow instrument equipped with a spinning band monochromator, at 23 °C (4 × 20 mm flow cell, 1.24-mm slits, 330–560 nm, 400 lines mm^−1^/500 nm gratings), as described earlier (28, 33, 58, 59, 60). When indicated, traces were collected in the OLIS “pretrigger mode,” indicating some of the final absorbances from the end of the previous shot in order to judge whether a reaction has gone to completion. Data from 3 to 8 shots were averaged and processed using OLIS GlobalWorks software (OLIS). Data were also transformed to Excel files and analyzed using GraphPad Prism (GraphPad) and KinTek Explorer software, utilizing an Apple computer operating system with macOS Catalina 10.15 software (macOS). SVD analysis was done within the GlobalWorks program, and files were also transferred to Excel and then to Apple programs.

## Data availability

All data are contained within the article and the supporting information.

## Supporting information

This article contains supporting information.

## Conflict of interest

The authors declare that they have no conflicts of interest with the contents of this article.
