# Supplementary material for: Stepwise binding of inhibitors to human cytochrome P450 17A1 and rapid kinetics of inhibition of androgen biosynthesis
Source: J Biol Chem. 2021 Jul 15;297(2):100969. doi: 10.1016/j.jbc.2021.100969 (PMC8350020; doi:10.1016/j.jbc.2021.100969)
Supplement: Supplemental Figures S1–S8 and Table S1 [file mmc1.docx]

**Supporting Information**

**Stepwise binding of inhibition to human cytochrome P450 17A1 and rapid kinetics of inhibition of androgen biosynthesis**

F. Peter Guengerich, Kevin D. McCarty, Jesse G. Chapman, and Yasuhiro Tateishi

Department of Biochemistry, Vanderbilt University School of Medicine, Nashville, TN 37232-0146 U.S.A.

Table of Contents

Table S1. IC_50_ values for inhibitors.

Figure S1. Derivatization of 17α-OH progesterone and DHEA.

Figure S2. UPLC of progesterone, 17α-OH progesterone, 17α-OH pregnenolone, and DHEA dansylhydrazones.

Figure S3. Mass spectra of dansylhydrazones.

Figure S4. ^1^H-NMR spectra of di-dansyl hydrazone of 17α-OH progesterone.

Figure S5. Examples of UPLC chromatograms for formation of products.

Figure S6. Linearity of mass spectral responses of dansyl hydrazine derivatives.

Figure S7. Linearity of product formation as a function of P450 concentration using bacterial membranes (Bactosomes®).

Figure S8. Dependence of progesterone 17α-hydroxylation and 17α-OH pregnenolone lyase activities on *b*_5_ concentration with bacterial membranes (Bactosomes®).

**Table S1**

**IC_50_ values for inhibitors (from this study and previous literature)**

|  |  |  | IC_50_, µM |  |
| --- | --- | --- | --- | --- |
| Reference |  | Progesterone 17α-hydroxylation | 17α-OH Pregnenolone➝DHEA  (lyase) | Ratio  (17-hydroxylation/  lyase) |
| This work (Fig. 3, Table 1) | Ketoconazole | 0.087 | 0.23 | 0.38 |
|  | Clotrimazole | 0.060 | 0.099 | 0.61 |
|  | Abiraterone | 0.0032 | 0.0042 | 0.76 |
|  | (*S*)-Orteronel | 0.42 | 1.06 | 0.40 |
|  | (*S*)-Seviteronel | 3.5 | 3.4 | 1.0 |
|  |  |  |  |  |
| (1) | (*S*)-Orteronel | 0.32 | 0.49 | 1.3 |
|  | (*R*)-Orteronel | 2.1 | 2.8 | 0.75 |
|  |  |  |  |  |
| (2) | Galaterone | 0.13 | 0.10 | 1.3 |
|  | Abiraterone | 0.076 | 0.036 | 2.1 |
|  | (*S*)-Orteronel | 0.95 | 0.210 | 4.5 |
|  | (*R*)-Orteronel | 4.00 | 0.46 | 8 |
|  | (*S*)-Seviteronel | 1.17 | 0.43 | 2.7 |
|  | (*R*)-Seviteronel | 11.3 | 5.0 | 2.3 |
|  |  |  |  |  |
| (3) | (*S*)-Orteronel | 0.76 | 0.14 | 5.4 |
|  | (*S*)-Orteronel (cells) | 0.038 | 0.027 | 1.4 |
|  |  |  |  |  |
| (4) | *S*-Seviteronel | 0.69 | 0.069 | 10 |
|  |  |  |  |  |
| (5) | Clotrimazole | 0.18 | 0.056 | 3.3 |
|  | Ketoconazole | 0.136 | 0.063 | 2.2 |
|  |  |  |  |  |
| Other studies (2) | Abiraterone | 1.5-72 | 0.0029-0.80 | 0.1-1.4 |

**Figure S1. Derivatization of 17α-OH progesterone and DHEA** (6, 7).

**Figure S2. UPLC of progesterone, 17α-OH progesterone, 17α-OH pregnenolone, and DHEA dansylhydrazones.** *A*, progesterone (*t*_R_ 9.51, 9.81 min) and 17α-OH progesterone (*t*_R_ 8.01, 8.44 min); *B*, pregnenolone (*t*_R_ 8.68 min) and DHEA (*t*_R_ 7.86 min). Detection was using fluorescence (F_332/516_ , arbitrary EU units).

**Figure S3.** **Mass spectra of dansylhydrazones.** *A*, 17α-OH pregnenlolone; *B*, DHEA; *C*, progesterone; *D*, 17α-OH progesterone.

**Figure S4. ^1^H-NMR spectra of di-dansyl hydrazone of 17α-OH progesterone.** To a solution of 17α-OH progesterone (33 mg, 0.10 mmol) in CH_3_OH (10 ml) were added dansyl hydrazine (265 mg, 1.0 mmol) and CF_3_CO_2_H (3.8 μl, 0.05 mmol) and the mixture was stirred at room temperature for 15 h. Water was added, and the resulting mixture was raised to pH 10 with NaOH and then extracted with ethyl acetate. The organic layer was washed with brine, dried over anhydrous Na_2_SO_4_, and evaporated to dryness. The residue was purified with silica gel column chromatography (*n-*hexane/ethyl acetate, 2/1→1/1→1/2, v/v) to obtain a yellow solid (76 mg, 92% yield) as a mixture of *E*/*Z* isomers, as judged by UPLC. The product was analyzed by ^1^H NMR (CDCl_3_, 600 MHz, tetramethylsilane (δ=0) used as an internal standard). Peaks for residual ethyl acetate are present: δ 1.3-CH_2_*CH_3_*, 2.1- CO*CH_3_*, 4.2- *CH_2_*CH_3_. *Inset*, The peaks of interest are the H-4 protons (A ring) of 17α-OH progesterone at δ 5.73 and 6.07, in a 2-1 ratio for the *E*- and *Z*-isomers (not assigned to individual isomers).

**Figure S5. Examples of UPLC chromatograms for formation of products.** Residual substrates are also shown. *A*, 17α-OH pregnenolone; *B*, DHEA; *C*, progesterone; *D*, 17α-OH progesterone. The first set of doublets (*t*_R_ 5.5-6.0 min) in the 17-OH progesterone chromatogram (Part *D*) corresponds to 16α-OH progesterone and the second set (*t*_R_ 6.2-6.6 min) corresponds to 17α-OH progesterone. The chromatography conditions differ from those used in Fig. S2. In Part *B*, the incubation was done with 20 nM P450 17A1 for 5 min at 37 °C to obtain the product trace shown. In Part *D*, the incubation was done with 250 nM P450 17A1 for 3 min at 23 °C to obtain the product trace shown.

**Figure S6. Linearity of mass spectral responses of dansyl hydrazine derivatives.** *A*, 17α-OH pregnenolone; *B*, DHEA; *C*, progesterone; *D*, 17α-OH progesterone. All assays involved positive ion electrospray.

**Figure S7. Linearity of progesterone 17α-hydroxylation as a function of P450 concentration using bacterial membranes (Bactosomes®).** No *b*_5_ was added. The reaction time was 5 min (at 37 °C).

**Figure S8. Dependence of progesterone 17α-hydroxylation and 17α-OH pregnenolone lyase activities on *b*_5_ concentration with bacterial membranes (Bactosomes®).** *A*, progesterone 17α-hydroxylation; *B*, 17α-OH pregnenolone lyase activity. Both fits are to hyperbolic curves (Prism) beginning at the y values corresponding to no *b*_5_ added.

References (for Supporting Information section only)

1. Gonzalez, E., and Guengerich, F. P. (2017) Kinetic processivity of the two-step oxidations of progesterone and pregnenolone to androgens by human cytochrome P450 17A1. *J. Biol. Chem.* **292**, 13168-13185

2. Petrunak, E. M., Rogers, S. A., Aube, J., and Scott, E. E. (2017) Structural and functional evaluation of clinically relevant inhibitors of steroidogenic cytochrome P450 17A1. *Drug Metab. Dispos.* **45**, 635-645

3. Yamaoka, M., Hara, T., Hitaka, T., Kaku, T., Takeuchi, T., Takahashi, J., Asahi, S., Miki, H., Tasaka, A., and Kusaka, M. (2012) Orteronel (TAK-700), a novel non-steroidal 17,20-lyase inhibitor: effects on steroid synthesis in human and monkey adrenal cells and serum steroid levels in cynomolgus monkeys. *J. Steroid Biocheem. Mol. Biol.* **129**, 115-128

4. Rafferty, S. W., Eisner, J. R., Moore, W. R., Schotzinger, R. J., and Hoekstra, W. J. (2014) Highly-selective 4-(1,2,3-triazole)-based P450c17a 17,20-lyase inhibitors. *Bioorg. Med. Chem. Lett.* **24**, 2444-2447

5. Munkboel, C. H., Rasmussen, T. B., Elgaard, C., Olesen, M.-L. K., Kretschmann, A. C., and Styrishave, B. (2019) The classic azole antifungal drugs are highly potent endocrine disruptors in vitro inhibiting steroidogenic CYP enzymes at concentrations lower than therapeutic C_max_. *Toxicology* **425**, 152247

6. Chayen, R., Dvir, R., Gould, S., and Harell, A. (1971) 1-Dimethylaminonaphthalene-5-sulfonyl hydrazine (dansyl hydrazine): a fluorometric reagent for carbonyl compounds. *Anal. Biochem.* **42**, 283-286

7. Appelblad, P., Pontén, E., Jaegfeldt, H., Bäckström, T., and Irgum, K. (1997) Derivatization of steroids with dansylhydrazine using trifluoromethanesulfonic acid as catalyst. *Anal. Chem.***69**, 4905-4911
